# MicroRNA's role as biomarkers of lupus nephritis in children

**DOI:** 10.1186/1546-0096-12-S1-P107

**Published:** 2014-09-17

**Authors:** Khalid Abulaban, Ndate Fall, Shannen Nelson, David Witte, Prasad Devarajan, Michael Bennett, Hermine I Brunner

**Affiliations:** 1 Cincinnati Children's Hospital and Research Center, Cincinnati, USA

## Introduction

There is a dire need of non-invasive biomarkers of lupus nephritis (LN) activity. MicroRNAs (miRNAs) are endogenous, non-coding, single-stranded RNAs involved in the regulation of host genome expression at the post-transcriptional level. Previous miRNA expression profiling studies have generated some unique miRNA signatures (including miR-125a, miR-127, miR-146a,miR-150,miR-155) that are associated with systemic lupus erythematosus (SLE), but their role as biomarkers of LN has not been well examined.

## Objectives

To determine levels of candidate miRNA biomarkers in blood and urine to the assess the differences in children with active LN vs. extrarenal SLE vs. controls. Also to assess potential associations with the LN-Panel [NGAL, MCP1, transferrin (Tf), Cystatin C, Beta-trace protein] of novel urinary biomarkers and traditional LN biomarkers (GFR, protein: creatinine ratio); and to explore combinations of biomarkers to predict LN activity using stepwise regression modeling.

## Methods

In this ongoing research study, miRNA was measured using rtPCR in the urine pellet (UP) and supernatant (sup) as well as blood in patients with JIA and fibromyalgia serving as disease controls and 14 patients with active LN and 10 with extrarenal SLE every 6 months. Disease activity was measured by the SLEDAI. Urine samples were assayed for the LN-Panel and traditional biomarkers were recorded.

## Results

LN activity measured by the SLEDAI was strongly positively correlated with sup levels of miR-125a (r > 0.7), moderately with miR-127, miR-150, miR-155 (r> 0.5), and moderately with miR-146a in blood cells. In the sup, miRNA-125a was significantly higher with active vs. not active LN (WSR test; p= 0.018) and was moderately associated with all LN-Panel markers (r = 0.53 – 0.66). Exploratory regression modeling suggests that NGAL, MCP1, TF and miR-125a in the sup are relevant combinatorial LN biomarkers (all standardized beta coefficients > |0.3| and p < 0.01) to predict LN activity (renal SLEDAI) at high accuracy (R-square = 87%). Similar results were found when the renal BILAG or SLICC Renal Activity Score were used to measure LN activity instead of the renal SLEDAI.

## Conclusion

The miRNAs miR-125a, miR-127, miR-146a,miR-150,miR-155 are differentially excreted in the urine with active LN. Based on initial evaluations these miRNA complement newer LN biomarkers (NGAL, MCP, TF) in their ability to assess concurrent LN activity.

## Disclosure of interest

None declared.

